# Distribution Committee's Report and Awards

**Published:** 1917-08-11

**Authors:** 


### METROPOLITAN HOSPITAL SUNDAY FUND.

### Distribution Committee's Report and Awards.

WE publish below the report of the Committee of Distribution of the Metropolitan Hospital Sunday Fund and the awards for the year 1917. The meeting of the Council of the Fund to sanction these awards is being held as we go to press.

### The Committee's Report.

THE Committee of Distribution held their first meeting on Tuesday, May 29, and then and at subsequent meetings examined the reports and income and expenditure accounts of the several institutions seeking to participate, and made their awards in conformity with the Laws of the Constitution.

The Committee beg to report that the total of the Fund on August 8 will amount to £70,500.

This year 241 institutions have applied to participate in the Fund, being seven less than in 1916, and the Committee now recommend the distribution of £70,061 16s. 6d. to the several hospitals, institutions, dispensaries and nursing associations shown in the appendix hereto.

The Committee having reduced the bases of award to seven institutions, the governing bodies of the same were informed thereof in accordance with the Laws of the Constitution. Six deputations attended the Committee, and after hearing their several explanations, the bases of award were finally determined.

Seven and a-half per cent. of the total sum available for distribution is appropriated to the purchase of surgical appliances during the ensuing year, and 2½ per cent. for district nursing associations.

In compliance with an order of the Council, statistics have been prepared as usual for the use of its members, showing the number of beds in hospitals, the cost of patients in hospitals and dispensaries, the proportionate expense of management to maintenance, and other information. A copy thereof has been sent to each member of the Council.

W. H. DUNN, Lord Mayor, President and Treasurer.
C. ERNEST TRITTON, Vice-Chairman.
WILLIAM S. CHURCH, Chairman of Committee.
W. HARDY HARWOOD. JOHN C. BELL.
ADRIAN POLLOCK. SOMERLEYTON.
F. A. BEVAN. MARCUS SAMUEL.
G. H. MAKINS.
JOHN HENRY BUXTON, } Hon.
C. C. WAKEFIELD. } Secs.

The Mansion House, July 26, 1917.

